# *tert*-Butyl *N*-(4-methyl-2-pyrid­yl)­carbamate

**DOI:** 10.1107/S1600536808032327

**Published:** 2008-10-31

**Authors:** Pierre Koch, Dieter Schollmeyer, Stefan Laufer

**Affiliations:** a Institute of Pharmacy, Department of Pharmaceutical and Medicinal Chemistry, Eberhard-Karls-University Tübingen, Auf der Morgenstelle 8, 72076 Tübingen, Germany; b Department of Organic Chemistry, Johannes Gutenberg-University Mainz, Duesbergweg 10-14, D-55099 Mainz, Germany

## Abstract

The crystal structure of the title compound, C_11_H_16_N_2_O_2_, contains two crystallographically independent mol­ecules forming dimers by pairs of inter­molecular N—H⋯N hydrogen bonds. The two mol­ecules are related by a pseudo-twofold axis. The dihedral angle between the pyridine ring and the carbamate plane differs in the two mol­ecules [12.1 (3) and 3.5 (3)°].

## Related literature

For the preparation of the title compound, see: Laufer & Koch (2008 ▶); Koch *et al.* (2008 ▶). For applications of functionalized 2-amino­pyridines, see, for example: Peifer *et al.* (2006 ▶); Kuo, DeAngelis *et al.* (2005 ▶); Kuo, Wang *et al.* (2005 ▶); Swahn *et al.* (2006 ▶).
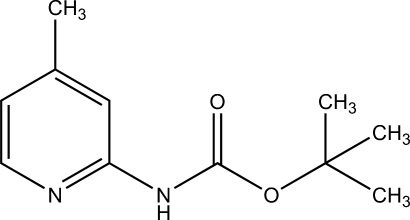


## Experimental

### 

#### Crystal data

C_11_H_16_N_2_O_2_
*M* *_r_* = 208.26
Orthorhombic, 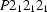

*a* = 10.5850 (6) Å
*b* = 11.6854 (6) Å
*c* = 18.5568 (15) Å
*V* = 2295.3 (3) Å^3^
*Z* = 8
Cu *K*α radiation
μ = 0.68 mm^−1^
*T* = 193 (2) K
0.51 × 0.16 × 0.06 mm

#### Data collection

Enraf–Nonius CAD-4 diffractometer
Absorption correction: none
4711 measured reflections
2471 independent reflections
1782 reflections with *I* > 2σ(*I*)
*R*_int_ = 0.061
3 standard reflections frequency: 60 min intensity decay: 3%

#### Refinement

*R*[*F* ^2^ > 2σ(*F* ^2^)] = 0.057
*wR*(*F* ^2^) = 0.149
*S* = 1.01
2471 reflections
280 parameters
H-atom parameters constrained
Δρ_max_ = 0.25 e Å^−3^
Δρ_min_ = −0.25 e Å^−3^

### 

Data collection: *CAD-4 Software* (Enraf–Nonius, 1989 ▶); cell refinement: *CAD-4 Software*; data reduction: *CORINC* (Dräger & Gattow, 1971 ▶); program(s) used to solve structure: *SIR97* (Altomare *et al.*, 1999 ▶); program(s) used to refine structure: *SHELXL97* (Sheldrick, 2008 ▶); molecular graphics: *PLATON* (Spek, 2003 ▶); software used to prepare material for publication: *PLATON*.

## Supplementary Material

Crystal structure: contains datablocks I, global. DOI: 10.1107/S1600536808032327/bt2808sup1.cif

Structure factors: contains datablocks I. DOI: 10.1107/S1600536808032327/bt2808Isup2.hkl

Additional supplementary materials:  crystallographic information; 3D view; checkCIF report

## Figures and Tables

**Table 1:** Hydrogen-bond geometry (Å, °)

*D*—H⋯*A*	*D*—H	H⋯*A*	*D*⋯*A*	*D*—H⋯*A*
N8*A*—H8*A*⋯N2*B*	0.94	2.05	2.980 (5)	171
N8*B*—H8*B*⋯N2*A*	0.98	2.04	3.015 (5)	173

**Fig. 1.**
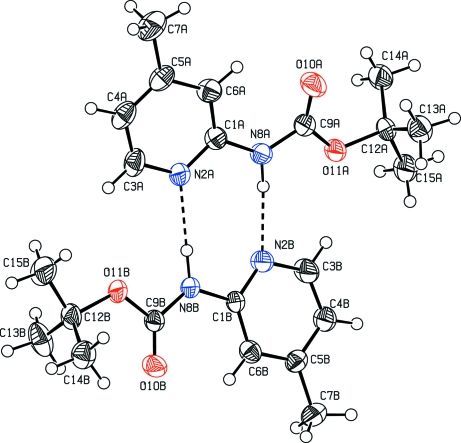
View of compound I. Displacement ellipsoids are drawn at the 50% probability level. H atoms are depicted as circles of arbitrary size. Hydrogen bonds are drawn as dashed lines.

## supplementary crystallographic information

### Comment

N-substituted 2-aminopyridin-4-yl derivatives can be found in different biological active compounds, like p38 MAP kinase inhibitors (Peifer *et al.*, 2006), VEGFR-2 inhibitors (Kuo, Wang *et al.*, 2005*a*), CDK inhibitors (Kuo, DeAngelis *et al.*, 2005) or JNK3 inhibitors (Swahn *et al.*, 2006). The title compound, *tert*-butyl 4-methylpyridin-2-ylcarbamate (I), was synthesized as an intermediate in the synthesis of 2-alkylsulfanyl-5-(2-aminopyridin-4-yl)-4-(4-fluorophenyl)imidazoles as potent p38 MAP kinase inhibitors (Laufer & Koch, 2008; Koch *et al.*, 2008).

The crystal stucture of the title compound I, Fig. 1, contains two crystallographically independent molecules forming dimers by intermolecular N–H···N hydrogen bonds. The two molecules are related by a pseudo 2-fold axis.

As might be expected the pyridine ring as well as the carbamate fragment are planar. The dihedral angle between the pyridine ring and the carbamate plane of molecule A [12.1 (3)°] is bigger than in molecule B [3.5 (3)°].

The N8—C9 is shorter than a normal N—C-bond and longer than a N-C-bond (N8A—C9A: 1.373 (6) Å; N8B—C9B: 1.367 (5) Å), indicating the partially double bond character of the N8—C9-bond of the carbamate.

### Experimental

To a solution of freshly distilled *tert*-butanol (450 ml) and di-*tert*-butyl dicarbonate (16.81 g, 77.0 mmol) was added slowly 2-amino-4-methylpyridine (7.57 g, 70.0 mmol). The mixture was stirred at room temperature for 3 d, the solvent was removed *in vacuo* and the residue was recrystallized from hot 2-propanol, affording 12.30 g (84%) of I as colourless crystals (Laufer & Koch, 2008).

### Refinement

In the absence of significant anomalous dispersion effects, Friedel pairs were averaged. H-atom bonded to N were located from a difference Fourier map and constrained to this position. All hydrogen atoms bonded to C  were placed at calculated positions with C—H = 0.95 Å (for aromatic C) or 0.98 Å (for *sp*^3^ C-atoms) and  refined in the riding-model approximation with isotropic displacement parameters  set to 1.2 (1.5 for methyl groups) times of the *U*_eq_ of the parent atom.

### Crystal data

**Table:**

C_11_H_16_N_2_O_2_	*F*(000) = 896
*M**_r_* = 208.26	*D*_x_ = 1.205 Mg m^−^^3^
Orthorhombic, *P*2_1_2_1_2_1_	Cu *K*α radiation, λ = 1.54178 Å
Hall symbol: P 2ac 2ab	Cell parameters from 25 reflections
*a* = 10.5850 (6) Å	θ = 21–26°
*b* = 11.6854 (6) Å	µ = 0.68 mm^−^^1^
*c* = 18.5568 (15) Å	*T* = 193 K
*V* = 2295.3 (3) Å^3^	Plate, colourless
*Z* = 8	0.51 × 0.16 × 0.06 mm

### Data collection

**Table:**

Enraf–Nonius CAD-4 diffractometer	*R*_int_ = 0.061
Radiation source: rotating anode	θ_max_ = 70.0°, θ_min_ = 4.5°
graphite	*h* = −12→12
ω/2θ scans	*k* = −13→14
4711 measured reflections	*l* = −22→22
2471 independent reflections	3 standard reflections every 60 min
1782 reflections with *I* > 2σ(*I*)	intensity decay: 3%

### Refinement

**Table:**

Refinement on *F*^2^	Secondary atom site location: difference Fourier map
Least-squares matrix: full	Hydrogen site location: inferred from neighbouring sites
*R*[*F*^2^ > 2σ(*F*^2^)] = 0.057	H-atom parameters constrained
*wR*(*F*^2^) = 0.149	*w* = 1/[σ^2^(*F*_o_^2^) + (0.0707*P*)^2^] where *P* = (*F*_o_^2^ + 2*F*_c_^2^)/3
*S* = 1.01	(Δ/σ)_max_ = 0.002
2471 reflections	Δρ_max_ = 0.25 e Å^−^^3^
280 parameters	Δρ_min_ = −0.25 e Å^−^^3^
0 restraints	Extinction correction: SHELXL97 (Sheldrick, 2008), Fc^*^=kFc[1+0.001xFc^2^λ^3^/sin(2θ)]^-1/4^
Primary atom site location: structure-invariant direct methods	Extinction coefficient: 0.0021 (4)

### Special details

**Table:**

Geometry. All e.s.d.'s (except the e.s.d. in the dihedral angle between two l.s. planes) are estimated using the full covariance matrix. The cell e.s.d.'s are taken into account individually in the estimation of e.s.d.'s in distances, angles and torsion angles; correlations between e.s.d.'s in cell parameters are only used when they are defined by crystal symmetry. An approximate (isotropic) treatment of cell e.s.d.'s is used for estimating e.s.d.'s involving l.s. planes.
Refinement. Friedel pairs merged. Refinement of *F*^2^ against ALL reflections. The weighted *R*-factor *wR* and goodness of fit *S* are based on *F*^2^, conventional *R*-factors *R* are based on *F*, with *F* set to zero for negative *F*^2^. The threshold expression of *F*^2^ > σ(*F*^2^) is used only for calculating *R*-factors(gt) *etc*. and is not relevant to the choice of reflections for refinement. *R*-factors based on *F*^2^ are statistically about twice as large as those based on *F*, and *R*- factors based on ALL data will be even larger.

### Fractional atomic coordinates and isotropic or equivalent isotropic displacement parameters (Å^2^)

**Table:**

	*x*	*y*	*z*	*U*_iso_*/*U*_eq_	
C1A	0.0792 (4)	0.7774 (3)	0.3417 (2)	0.0335 (10)	
N2A	0.1189 (4)	0.7180 (3)	0.28496 (19)	0.0364 (8)	
C3A	0.0776 (5)	0.6104 (4)	0.2792 (3)	0.0451 (12)	
H3A	0.1052	0.5663	0.2392	0.054*	
C4A	−0.0032 (5)	0.5596 (4)	0.3282 (3)	0.0462 (12)	
H4A	−0.0309	0.4830	0.3215	0.055*	
C5A	−0.0430 (4)	0.6223 (4)	0.3872 (3)	0.0407 (11)	
C6A	0.0026 (4)	0.7332 (4)	0.3947 (2)	0.0393 (11)	
H6A	−0.0189	0.7777	0.4357	0.047*	
C7A	−0.1333 (6)	0.5727 (5)	0.4407 (3)	0.0613 (15)	
H7A	−0.1060	0.4952	0.4536	0.092*	
H7B	−0.2180	0.5696	0.4195	0.092*	
H7C	−0.1349	0.6207	0.4840	0.092*	
N8A	0.1233 (4)	0.8913 (3)	0.3411 (2)	0.0382 (9)	
H8A	0.1733	0.9180	0.3026	0.046*	
C9A	0.0976 (4)	0.9746 (4)	0.3912 (2)	0.0379 (11)	
O10A	0.0460 (4)	0.9595 (3)	0.44786 (18)	0.0545 (10)	
O11A	0.1393 (3)	1.0749 (2)	0.36505 (16)	0.0373 (7)	
C12A	0.1276 (4)	1.1805 (4)	0.4084 (2)	0.0370 (10)	
C13A	0.2094 (5)	1.1704 (5)	0.4741 (3)	0.0490 (12)	
H13A	0.2964	1.1536	0.4595	0.073*	
H13B	0.1778	1.1084	0.5048	0.073*	
H13C	0.2074	1.2426	0.5009	0.073*	
C14A	−0.0098 (5)	1.2060 (4)	0.4244 (3)	0.0497 (13)	
H14A	−0.0440	1.1466	0.4562	0.075*	
H14B	−0.0578	1.2074	0.3793	0.075*	
H14C	−0.0165	1.2807	0.4482	0.075*	
C15A	0.1797 (5)	1.2711 (4)	0.3565 (3)	0.0512 (13)	
H15A	0.1313	1.2694	0.3115	0.077*	
H15B	0.2687	1.2548	0.3463	0.077*	
H15C	0.1724	1.3469	0.3786	0.077*	
C1B	0.3724 (4)	0.8850 (3)	0.1817 (2)	0.0294 (9)	
N2B	0.2977 (4)	0.9520 (3)	0.2209 (2)	0.0382 (9)	
C3B	0.3289 (5)	1.0633 (4)	0.2247 (3)	0.0447 (12)	
H3B	0.2760	1.1129	0.2518	0.054*	
C4B	0.4328 (5)	1.1091 (4)	0.1916 (2)	0.0413 (11)	
H4B	0.4524	1.1879	0.1972	0.050*	
C5B	0.5093 (4)	1.0392 (4)	0.1496 (2)	0.0357 (10)	
C6B	0.4761 (4)	0.9242 (4)	0.1449 (2)	0.0348 (10)	
H6B	0.5250	0.8732	0.1163	0.042*	
C7B	0.6208 (5)	1.0861 (4)	0.1101 (3)	0.0491 (12)	
H7D	0.5975	1.1007	0.0598	0.074*	
H7E	0.6900	1.0305	0.1117	0.074*	
H7F	0.6479	1.1577	0.1328	0.074*	
N8B	0.3330 (4)	0.7691 (3)	0.18270 (19)	0.0336 (8)	
H8B	0.2592	0.7567	0.2135	0.040*	
C9B	0.3881 (4)	0.6812 (4)	0.1456 (2)	0.0313 (9)	
O10B	0.4763 (3)	0.6874 (3)	0.10641 (17)	0.0434 (8)	
O11B	0.3214 (3)	0.5851 (2)	0.16178 (16)	0.0377 (7)	
C12B	0.3526 (4)	0.4770 (4)	0.1243 (3)	0.0406 (11)	
C13B	0.3232 (6)	0.4884 (5)	0.0454 (3)	0.0589 (15)	
H13D	0.3829	0.5418	0.0231	0.088*	
H13E	0.2369	0.5175	0.0395	0.088*	
H13F	0.3304	0.4134	0.0221	0.088*	
C14B	0.4870 (5)	0.4394 (4)	0.1387 (3)	0.0481 (12)	
H14D	0.5003	0.4327	0.1908	0.072*	
H14E	0.5457	0.4962	0.1189	0.072*	
H14F	0.5021	0.3651	0.1158	0.072*	
C15B	0.2625 (5)	0.3941 (4)	0.1610 (4)	0.0652 (17)	
H15D	0.1752	0.4186	0.1524	0.098*	
H15E	0.2793	0.3932	0.2129	0.098*	
H15F	0.2749	0.3171	0.1412	0.098*	

### Atomic displacement parameters (Å^2^)

**Table:**

	*U*^11^	*U*^22^	*U*^33^	*U*^12^	*U*^13^	*U*^23^
C1A	0.036 (2)	0.024 (2)	0.040 (2)	0.0047 (18)	0.0011 (19)	0.0019 (19)
N2A	0.0373 (19)	0.0282 (18)	0.0438 (19)	−0.0015 (16)	0.0037 (17)	−0.0048 (16)
C3A	0.045 (3)	0.026 (2)	0.064 (3)	−0.003 (2)	−0.006 (2)	−0.004 (2)
C4A	0.042 (3)	0.030 (2)	0.067 (3)	−0.007 (2)	−0.003 (2)	0.008 (2)
C5A	0.038 (2)	0.034 (2)	0.050 (3)	−0.005 (2)	−0.005 (2)	0.013 (2)
C6A	0.042 (3)	0.034 (2)	0.042 (2)	0.002 (2)	0.005 (2)	0.005 (2)
C7A	0.066 (4)	0.052 (3)	0.066 (3)	−0.021 (3)	0.001 (3)	0.017 (3)
N8A	0.046 (2)	0.0258 (18)	0.043 (2)	−0.0051 (17)	0.0153 (19)	−0.0017 (16)
C9A	0.041 (3)	0.033 (2)	0.039 (2)	−0.006 (2)	0.008 (2)	−0.0046 (19)
O10A	0.076 (3)	0.0417 (19)	0.0456 (18)	−0.0087 (19)	0.0239 (19)	−0.0056 (16)
O11A	0.0443 (18)	0.0245 (15)	0.0431 (16)	−0.0019 (14)	0.0098 (14)	−0.0037 (13)
C12A	0.041 (2)	0.025 (2)	0.045 (2)	0.001 (2)	0.006 (2)	−0.011 (2)
C13A	0.048 (3)	0.048 (3)	0.051 (3)	0.004 (2)	−0.003 (2)	−0.008 (3)
C14A	0.040 (3)	0.046 (3)	0.063 (3)	0.007 (2)	0.003 (2)	−0.016 (2)
C15A	0.066 (3)	0.024 (2)	0.063 (3)	−0.004 (2)	0.012 (3)	−0.005 (2)
C1B	0.033 (2)	0.0230 (19)	0.032 (2)	0.0001 (18)	0.0006 (18)	−0.0032 (16)
N2B	0.041 (2)	0.0272 (18)	0.046 (2)	−0.0003 (16)	0.0107 (17)	−0.0017 (17)
C3B	0.055 (3)	0.022 (2)	0.057 (3)	0.003 (2)	0.017 (3)	−0.002 (2)
C4B	0.049 (3)	0.029 (2)	0.046 (3)	−0.001 (2)	0.006 (2)	0.001 (2)
C5B	0.034 (2)	0.036 (2)	0.037 (2)	−0.0042 (19)	0.0002 (19)	0.0039 (19)
C6B	0.038 (2)	0.030 (2)	0.036 (2)	0.004 (2)	0.002 (2)	−0.0007 (18)
C7B	0.041 (3)	0.045 (3)	0.061 (3)	−0.011 (2)	0.010 (2)	−0.002 (2)
N8B	0.0341 (19)	0.0244 (17)	0.0422 (19)	−0.0048 (16)	0.0077 (16)	−0.0066 (15)
C9B	0.034 (2)	0.026 (2)	0.033 (2)	0.0006 (19)	0.0024 (19)	−0.0018 (18)
O10B	0.0500 (19)	0.0297 (16)	0.0503 (18)	0.0016 (15)	0.0171 (17)	−0.0035 (14)
O11B	0.0369 (16)	0.0251 (15)	0.0513 (18)	−0.0044 (13)	0.0061 (15)	−0.0104 (14)
C12B	0.039 (3)	0.028 (2)	0.055 (3)	0.005 (2)	−0.003 (2)	−0.011 (2)
C13B	0.070 (4)	0.047 (3)	0.060 (3)	0.022 (3)	−0.016 (3)	−0.020 (3)
C14B	0.047 (3)	0.041 (3)	0.056 (3)	0.001 (2)	−0.005 (2)	−0.002 (2)
C15B	0.056 (3)	0.031 (3)	0.108 (5)	−0.004 (3)	0.015 (3)	−0.016 (3)

### Geometric parameters (Å, °)

**Table:**

C1A—N2A	1.330 (5)	C1B—N2B	1.330 (5)
C1A—C6A	1.375 (6)	C1B—C6B	1.372 (6)
C1A—N8A	1.411 (5)	C1B—N8B	1.418 (5)
N2A—C3A	1.336 (5)	N2B—C3B	1.344 (5)
C3A—C4A	1.382 (7)	C3B—C4B	1.369 (6)
C3A—H3A	0.9500	C3B—H3B	0.9500
C4A—C5A	1.383 (7)	C4B—C5B	1.388 (6)
C4A—H4A	0.9500	C4B—H4B	0.9500
C5A—C6A	1.389 (6)	C5B—C6B	1.392 (6)
C5A—C7A	1.494 (7)	C5B—C7B	1.494 (6)
C6A—H6A	0.9500	C6B—H6B	0.9500
C7A—H7A	0.9800	C7B—H7D	0.9800
C7A—H7B	0.9800	C7B—H7E	0.9800
C7A—H7C	0.9800	C7B—H7F	0.9800
N8A—C9A	1.373 (5)	N8B—C9B	1.367 (5)
N8A—H8A	0.9418	N8B—H8B	0.9790
C9A—O10A	1.199 (5)	C9B—O10B	1.184 (5)
C9A—O11A	1.343 (5)	C9B—O11B	1.361 (5)
O11A—C12A	1.477 (5)	O11B—C12B	1.479 (5)
C12A—C13A	1.500 (6)	C12B—C13B	1.503 (7)
C12A—C14A	1.514 (7)	C12B—C14B	1.512 (7)
C12A—C15A	1.533 (6)	C12B—C15B	1.520 (7)
C13A—H13A	0.9800	C13B—H13D	0.9800
C13A—H13B	0.9800	C13B—H13E	0.9800
C13A—H13C	0.9800	C13B—H13F	0.9800
C14A—H14A	0.9800	C14B—H14D	0.9800
C14A—H14B	0.9800	C14B—H14E	0.9800
C14A—H14C	0.9800	C14B—H14F	0.9800
C15A—H15A	0.9800	C15B—H15D	0.9800
C15A—H15B	0.9800	C15B—H15E	0.9800
C15A—H15C	0.9800	C15B—H15F	0.9800
			
N2A—C1A—C6A	123.8 (4)	N2B—C1B—C6B	123.6 (4)
N2A—C1A—N8A	112.4 (4)	N2B—C1B—N8B	112.3 (4)
C6A—C1A—N8A	123.8 (4)	C6B—C1B—N8B	124.1 (4)
C1A—N2A—C3A	116.8 (4)	C1B—N2B—C3B	116.8 (4)
N2A—C3A—C4A	123.6 (5)	N2B—C3B—C4B	123.5 (4)
N2A—C3A—H3A	118.2	N2B—C3B—H3B	118.2
C4A—C3A—H3A	118.2	C4B—C3B—H3B	118.2
C3A—C4A—C5A	118.8 (4)	C3B—C4B—C5B	119.3 (4)
C3A—C4A—H4A	120.6	C3B—C4B—H4B	120.3
C5A—C4A—H4A	120.6	C5B—C4B—H4B	120.3
C4A—C5A—C6A	117.9 (4)	C4B—C5B—C6B	117.2 (4)
C4A—C5A—C7A	121.0 (4)	C4B—C5B—C7B	121.4 (4)
C6A—C5A—C7A	121.2 (5)	C6B—C5B—C7B	121.4 (4)
C1A—C6A—C5A	119.0 (4)	C1B—C6B—C5B	119.5 (4)
C1A—C6A—H6A	120.5	C1B—C6B—H6B	120.3
C5A—C6A—H6A	120.5	C5B—C6B—H6B	120.3
C5A—C7A—H7A	109.5	C5B—C7B—H7D	109.5
C5A—C7A—H7B	109.5	C5B—C7B—H7E	109.5
H7A—C7A—H7B	109.5	H7D—C7B—H7E	109.5
C5A—C7A—H7C	109.5	C5B—C7B—H7F	109.5
H7A—C7A—H7C	109.5	H7D—C7B—H7F	109.5
H7B—C7A—H7C	109.5	H7E—C7B—H7F	109.5
C9A—N8A—C1A	126.7 (4)	C9B—N8B—C1B	125.8 (4)
C9A—N8A—H8A	113.0	C9B—N8B—H8B	121.6
C1A—N8A—H8A	120.3	C1B—N8B—H8B	112.5
O10A—C9A—O11A	126.5 (4)	O10B—C9B—O11B	126.5 (4)
O10A—C9A—N8A	125.5 (4)	O10B—C9B—N8B	126.9 (4)
O11A—C9A—N8A	108.0 (3)	O11B—C9B—N8B	106.7 (3)
C9A—O11A—C12A	120.3 (3)	C9B—O11B—C12B	119.0 (3)
O11A—C12A—C13A	109.2 (4)	O11B—C12B—C13B	109.6 (4)
O11A—C12A—C14A	110.6 (4)	O11B—C12B—C14B	112.0 (4)
C13A—C12A—C14A	114.2 (4)	C13B—C12B—C14B	113.2 (4)
O11A—C12A—C15A	101.8 (3)	O11B—C12B—C15B	101.1 (3)
C13A—C12A—C15A	110.9 (4)	C13B—C12B—C15B	111.3 (5)
C14A—C12A—C15A	109.4 (4)	C14B—C12B—C15B	109.0 (4)
C12A—C13A—H13A	109.5	C12B—C13B—H13D	109.5
C12A—C13A—H13B	109.5	C12B—C13B—H13E	109.5
H13A—C13A—H13B	109.5	H13D—C13B—H13E	109.5
C12A—C13A—H13C	109.5	C12B—C13B—H13F	109.5
H13A—C13A—H13C	109.5	H13D—C13B—H13F	109.5
H13B—C13A—H13C	109.5	H13E—C13B—H13F	109.5
C12A—C14A—H14A	109.5	C12B—C14B—H14D	109.5
C12A—C14A—H14B	109.5	C12B—C14B—H14E	109.5
H14A—C14A—H14B	109.5	H14D—C14B—H14E	109.5
C12A—C14A—H14C	109.5	C12B—C14B—H14F	109.5
H14A—C14A—H14C	109.5	H14D—C14B—H14F	109.5
H14B—C14A—H14C	109.5	H14E—C14B—H14F	109.5
C12A—C15A—H15A	109.5	C12B—C15B—H15D	109.5
C12A—C15A—H15B	109.5	C12B—C15B—H15E	109.5
H15A—C15A—H15B	109.5	H15D—C15B—H15E	109.5
C12A—C15A—H15C	109.5	C12B—C15B—H15F	109.5
H15A—C15A—H15C	109.5	H15D—C15B—H15F	109.5
H15B—C15A—H15C	109.5	H15E—C15B—H15F	109.5
			
C6A—C1A—N2A—C3A	2.1 (6)	C6B—C1B—N2B—C3B	−1.2 (7)
N8A—C1A—N2A—C3A	−177.2 (4)	N8B—C1B—N2B—C3B	178.6 (4)
C1A—N2A—C3A—C4A	0.3 (7)	C1B—N2B—C3B—C4B	−1.0 (8)
N2A—C3A—C4A—C5A	−0.7 (7)	N2B—C3B—C4B—C5B	2.2 (8)
C3A—C4A—C5A—C6A	−1.2 (7)	C3B—C4B—C5B—C6B	−1.2 (7)
C3A—C4A—C5A—C7A	178.2 (5)	C3B—C4B—C5B—C7B	177.6 (5)
N2A—C1A—C6A—C5A	−4.0 (7)	N2B—C1B—C6B—C5B	2.0 (7)
N8A—C1A—C6A—C5A	175.3 (4)	N8B—C1B—C6B—C5B	−177.7 (4)
C4A—C5A—C6A—C1A	3.4 (7)	C4B—C5B—C6B—C1B	−0.8 (6)
C7A—C5A—C6A—C1A	−176.0 (5)	C7B—C5B—C6B—C1B	−179.5 (4)
N2A—C1A—N8A—C9A	178.9 (4)	N2B—C1B—N8B—C9B	177.5 (4)
C6A—C1A—N8A—C9A	−0.4 (7)	C6B—C1B—N8B—C9B	−2.7 (7)
C1A—N8A—C9A—O10A	9.6 (8)	C1B—N8B—C9B—O10B	−0.7 (7)
C1A—N8A—C9A—O11A	−169.8 (4)	C1B—N8B—C9B—O11B	179.9 (4)
O10A—C9A—O11A—C12A	2.3 (7)	O10B—C9B—O11B—C12B	−4.7 (6)
N8A—C9A—O11A—C12A	−178.3 (4)	N8B—C9B—O11B—C12B	174.8 (3)
C9A—O11A—C12A—C13A	65.5 (5)	C9B—O11B—C12B—C13B	−65.3 (5)
C9A—O11A—C12A—C14A	−61.0 (5)	C9B—O11B—C12B—C14B	61.2 (5)
C9A—O11A—C12A—C15A	−177.2 (4)	C9B—O11B—C12B—C15B	177.1 (4)

### Hydrogen-bond geometry (Å, °)

**Table:**

*D*—H···*A*	*D*—H	H···*A*	*D*···*A*	*D*—H···*A*
N8A—H8A···N2B	0.94	2.05	2.980 (5)	171
N8B—H8B···N2A	0.98	2.04	3.015 (5)	173
